# Self-rated health and perceived violence in the neighborhood is heterogeneous between young women and men

**DOI:** 10.1186/s12889-017-4969-1

**Published:** 2017-12-19

**Authors:** Aline Almeida Bentes, Cibele Comini César, César Coelho Xavier, Waleska Teixeira Caiaffa, Fernando Augusto Proietti

**Affiliations:** 1 Centro de Pesquisas René Rachou, Fundação Oswaldo Cruz, Av. Augusto de Lima 1715, Belo Horizonte, MG 30190-002 Brasil; 2 Federal University of Minas Gerais, Av. Prof. Alfredo Balena, 190, Belo Horizonte Cep, 30130-100 MG Brasil; 3 FASEH: Faculdade da Saúde e Ecologia Humana, Rua Sao Paulo, 958, Vespasiano, Cep 33200-000 MG Brasil

**Keywords:** Self-rated health, Perceived urban violence, Young adults (18–29 years), Urban population health

## Abstract

**Background:**

Self-rated health (SRH) is the general perception of an individual’s own health and a key indicator to measure health in population-based studies. Few studies have examined the association between perceived urban violence and SRH among young adults. There were an estimated 475,000 deaths in 2012 as a result of homicide on the world. Sixty percent of these deaths occurred among males aged 15–44 years, making homicide the third leading cause of death for this population group. This study aimed to determine and quantify the association between sex-specific perception of violence in the neighborhood and SRH among young adults.

**Methods:**

Participants included 955 young adults (18–29 years) residing in Belo Horizonte, Minas Gerais, Brazil between 2008 and 2009. Logistic regression analysis was used to estimate the strength of the associations. The perceived urban violence score was constructed from variables that assessed the respondents’ insecurity and perception of fear and danger of suffering some form of violence in the neighborhood using exploratory factor analysis.

**Results:**

18,3% of respondents rated their health as fair/ poor/very poor. Among women, fair/ poor/very poor SRH was associated with age between 25 and 29 years, low socioeconomic status score, being dissatisfied with weight, not exercising regularly, not having a healthy diet, and having some chronic disease. Men who rated their health as fair/poor/very poor more frequently smoked, were dissatisfied with their weight, did not exercise regularly, consumed fewer fruits and vegetables, and had some chronic disease compared to men who rated their health as very good/good. In the final model, after adjusting for confounding variables, perceived violence in the neighborhood was associated with poor SRH in young women only (OR = 1.52; 95% CI: 1.04–2.21).

**Conclusion:**

The results indicate that public and health policies should implement interventions on the neighborhood physical and social environment to improve the perception of safety and have a positive impact on people’s health, especially women.

## Background

Urban violence is one of the major causes of death and hospitalization among young adults (18-29 years) in several countries [1]. There were an estimated 475,000 deaths in 2012 as a result of homicide on the world. Sixty percent of these deaths occurred among males aged 15–44 years, making homicide the third leading cause of death for this population group [1]. Within low- and middle income countries, the highest estimated rate of homicide occurs in the Region of the Americas, with 28.5 homicides per 100,000 population, followed by the African Region with a rate of 10.9 homicides per 100,000 population [1]. Fatal violence is not evenly distributed among sex and age groups. The highest estimated rate of homicide in the world is found among males aged 15–29 years (18.2 per 100,000) [1].

In Brazil, the exposure to violence reveals a negative experience that has already affected an entire generation of young people: a recent survey by the Brazil Health Department indicated that 51.0% of young adults aged 18–29 years across all states and social strata in small, medium, and large cities have lost a close person in a violent way [2]. Nevertheless, deaths are only a fraction of the health and social burden arising from violence. In a nationally representative study of violence-related injury cases presenting at emergency departments during a 1-month period in Brazil, there were 4835 cases of violence related injury, of which 91% were victims of interpersonal violence and 9% were the result of self-directed violence. More than half of the victims (55%) were also young, aged 10–29 years [1].

Perceived urban violence has been generally defined as a negative emotional reaction to crime and includes reactions or attitudes such as avoiding public places, certain streets, going out at night, or engaging in leisure or sport activities in the neighborhood [3–5]. Structural characteristics such as physical and social disorder, low degree of social integration, urban segregation, and high crime rates in the neighborhood raise fear and anxiety levels among residents of certain urban areas leading to greater perceived violence and worse self-rated health (SRH) [3, 4].

SRH refers to the general perception of an individual’s own health and is one of the indicators most commonly used to measure the health of population groups in epidemiological studies [6–8]. Self-rating of health results from a cognitive process involving objective, subjective, and contextual aspects, i.e., even though it is an individual’s response, it is based on his/her physical, social, and cultural environment [6, 9]. Self-rated health has been identified by the American Institute of Medicine as one of the 20 key indicators to measure health in population-based studies [10]. In cohort studies, SRH is a strong predictor of morbidity and mortality [7, 8, 11].

Sex is one of several well-established independent determinants of SRH. In fact, women usually report worse SRH than men, especially at younger ages [12]. Consistently, a study conducted in Belo Horizonte, Minas Gerais, Brazil in 2013 evaluated the relationship between the physical and social environment and SRH in 4048 adults 18 years and older from a large urban center and showed that women were 38.0% more likely than men to rate their health as poor [12].

Nevertheless, knowledge about SRH in young adults (18–29 years) is still limited and to better understand the determinants of SRH in young adults it is important to examine their historical, socioeconomic and spatial context.

**Fig. 1:**
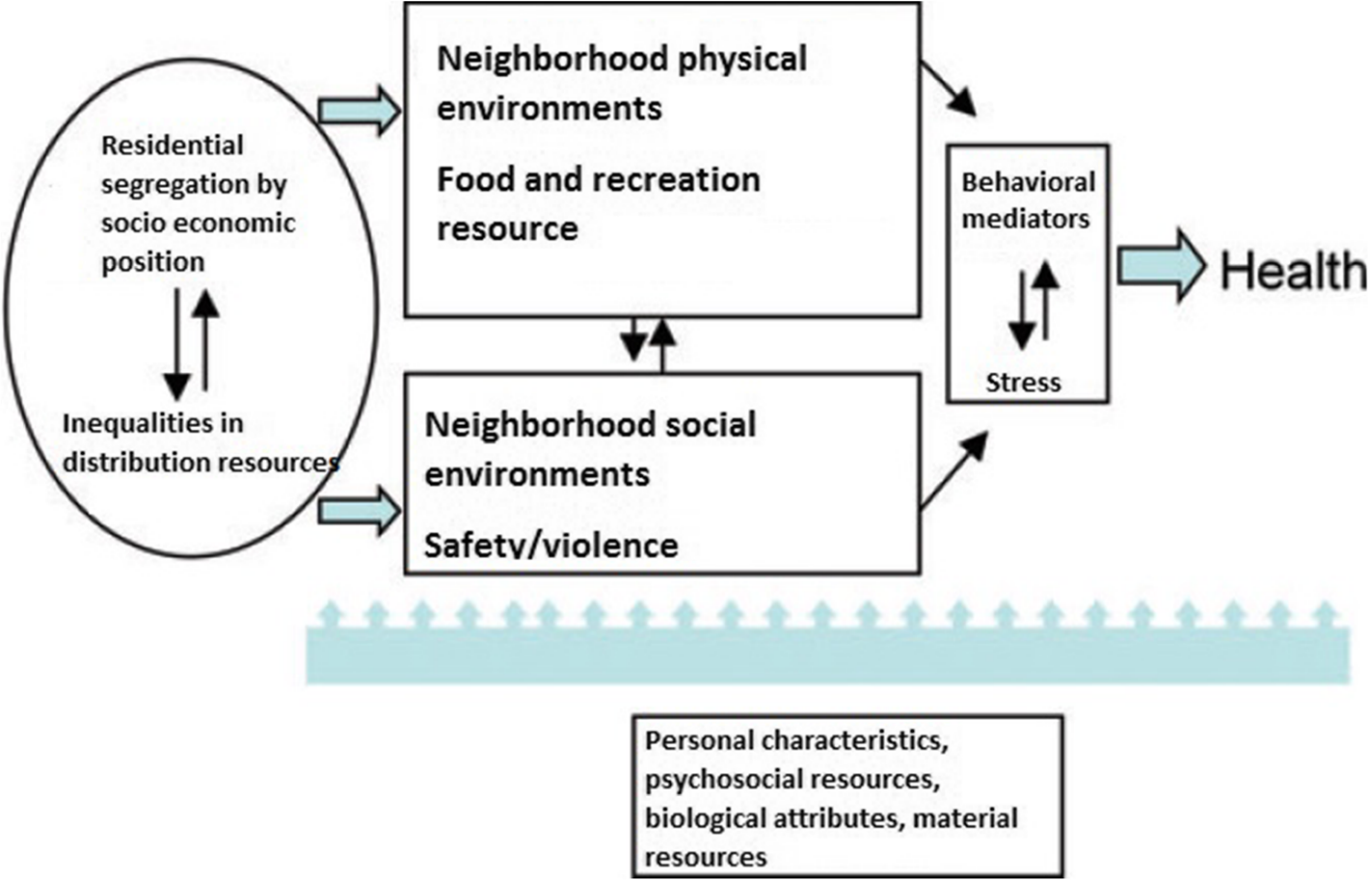
Schematic representation of the contribution of neighborhood environment to health, adapt from Diez Roux and Mair [13]

Diez-Roux and Mair in extensive literature review of neighborhood health proposes a theoretical model that describes how the physical and social characteristics of the neighborhood interrelate and affect people’s health [13]. Figure 1 summarizes how individual characteristics, behavioral mediators and stress also influence and are influenced by the physical environment and social aspects of neighborhood modifying health and SRH [13].

A study conducted in Illinois, USA, in 1995, which evaluated 2482 adults, found that residents of poor neighborhoods reported worse self-rated health, worse functional performance, and more chronic illnesses than neighborhood residents with greater advantages [14]. This association was mediated by the perception of physical disorganization such as abandoned buildings, noise, graffiti, vandalism, filth, disrepair and greater perception of fear in the neighborhood [14].

A survey made in 2004 with 1504 adults (18 years and over) were residing in Texas found that perceptions of neighborhood disorder may increase the risk of obesity by elevating levels of psychological distress, which, in turn, leads to chronic activation of the physiological stress response [15]. The citizens perceived the neighborhood as an unsafe, dirty and noisy place raised the levels of anxiety and depression of the residents and was associated with poorer quality in diet and obesity [15]. In this study not being satisfied with weight, not eating properly and not performing physical activities was also associated with worse self-rated health in young adults of both sexes.

A multi-center study conducted in six low- and middle-income countries consistently demonstrated that living in impoverished neighborhoods with greater social and physical disorganization where there is greater perception of urban violence is associated with greater psychological stress and numerous sleep problems [16]. Recent studies suggest that living in neighborhoods with greater physical and social disorganization is associated with poorer physical and mental health, poor self-rated health and depression [17–19]. It is possible that sleep quality is a mediating pathway that helps to explain the association between perceived urban violence and poor health.

A national health survey conducted in Denmark in 2000 with 12,028 adults (16+ years) evaluated the associations between violence, SRH and self-reported morbidity [20]. The authors observed that men aged 16–24 years were more likely to have experienced physical violence than women of the same age (OR = 3.20, 95% CI = 2.30–4.20). Female victims of physical violence were significantly more likely to rate their health as poor (OR = 2.02, 95% CI = 1.41–2.89) and to report anxiety (OR = 2.14, 95% CI = 1.35–3.37), depression (OR = 2.36, 95% CI = 1.55–3.60), and stomach ache (OR = 1.58, 95% CI = 1.01–2.47) than female non-victims. Associations between physical violence and poor self-rated health and self-reported morbidity were statistically associates for women, but not for men [20].

A longitudinal study of 8224 U.S. youths 12 to 18 years old at baseline reported that the risk for poor SRH was 4.6 times greater among subjects who were exposed to violence (OR = 4.63, 95% CI = 3.06–6.99) [21]. Having witnessed gun violence, being threatened at school, bullied repeatedly, or a victim of crime were independently and significantly associated with poor SRH. Additionally, the prevalence of fair/poor SRH was higher among female (56.0%), low-income (32.0%), and African-American youths (39.0%) [21].

The present study aimed to determine and quantify the association between sex specific perception of violence in the neighborhood and SRH among young adults. We hypothesized that there is heterogeneity in the impact of the perception of violence on self-rated health for men and women.

## Methods

This study assessed data from a population-based health survey conducted by the Belo Horizonte Urban Health Observatory (OSUBH) of the Federal University of Minas Gerais (UFMG) between 2008 and 2009 in two – Barreiro and Oeste – of the nine health districts of the city. Belo Horizonte (2.513.451 inhabitants) is the capital of Minas Gerais State located in southeast Brazil and the main city of the Belo Horizonte Metropolitan Area (5.873.841 inhabitants) [22]. The estimated population of each district is approximately 250,000.

The study area was divided into strata according to the health vulnerability index (HVI), developed by the Belo Horizonte City Health Department. The HIV is a summary measure that estimates the inequalities in the epidemiological profile of different social groups within the census tracts. It includes the following components: sanitation, housing, education, income, and health [22].

In each HVI stratum, selection was performed using a three-stage sampling methodology: census tract, address (residence), and resident (one adult). In total, 150 tracts were selected. Within each census tract, a simple random sample of household addresses registered in the database of the Municipality of Belo Horizonte was taken. Next, one adult resident (18 years or older) was drawn using a random number table.

At the end of the sampling process, 5.436 households had been selected. After being informed about the objectives of the study, residents who were drawn were invited to participate and sign a consent form. In total, 4.048 adults were interviewed with a refusal rate of 25.0%. For this study, we selected only young adults between the ages of 18 and 29 years, representing 955 participants. All participants answered a face-to-face questionnaire administered by trained interviewers. The questionnaire was composed of six modules: household, sociodemographic factors, health, mobility, social determinants of health, lifestyle and behaviors.

Detailed information about the SBH survey methodology can be found in Camargos et al. [23] and Friche et al. [22, 24].

The study was approved by the Research Ethics Committee of the Federal University of Minas Gerais (UFMG), Brazil, under protocol numbers ETCI 253/006 and ETCI 017/07.

### Variables

#### Response variable

Self-rated health was assessed by the question “In general, how would you rate your health?”, with five responses on a five-category scale: “very good”, “good”, “fair”, “poor”, or “very poor”. Responses were later categorized for analysis as fair/poor/very poor, and very good/good (reference category).

#### Variable of interest

The explanatory variable of interest was perceived urban violence, defined as a negative emotional reaction to crime, a social phenomenon that reduces social interaction and mutual trust among residents, causing a decline in the quality of life in the community or neighborhood [3–5].

The perceived urban violence score was constructed using the variables to assess the respondents’ perception of fear, danger, and insecurity of suffering some form of violence in the neighborhood. Participants were asked the following questions: What is the risk of being (1) personally threatened, robbed/mugged; (2) assaulted or threatened with aggression; (3) abducted (kidnapped); (4) hit by a stray bullet; (5) seriously injured or killed; and (6) a victim of police violence. Respondents rated the risk as very high, high, low, or very low. The perceived urban violence score was calculated using the principal components method and ranged from 1.21 to 4.85 (mean ± sd = 2.13 ± 0.69) for women and 1.21 to 4.65 (2.32 ± 0.74) for men.

Potential confounding variables were divided into the following categories:

A) Time residing in the neighborhood in years
B) Demographics: age (18–24 and 25–29 years); sex and marital status: married/living together or divorced/separated and single
C) Schooling: able to read/primary school equivalency; 1st-4th grades not completed; 1st-4th grades completed; 5th-8th grades not completed; 5th-8th grades completed; high school not completed; high school graduate/technical school/attended university; university graduate; post-undergraduate studies.
D) Socioeconomic: socioeconomic position score (SPS), detailed below;
E) Lifestyle and behaviors: 1) smoking: current smoker and non-smoker/former smoker; 2) alcohol consumption: non-drinker, moderate consumption (1–2 times a week and less than five drinks per day), or excessive consumption (≥ three times a week or more than five drinks per day); 3) consumption of fruits and vegetables: defined as consumption of at least one portion 5 days a week for the past 12 months, yes/no; 4) physical activity: physical activity during leisure time ≥ 30 min/day and physical activity during leisure time < 30 min/day (1% of respondents who performed physical activity exercised for <30 min/day).
F) Health condition: 1) whether he/she is satisfied with their own weight: yes/ no; 2) report of chronic disease: yes/no, detailed below;

The socioeconomic position score was constructed using 13 indicators: number of residents per bedroom; housing tenure (rented, owned, loaned, other); and presence or absence (yes/no) of the following items in the household: DVD player, videocassette recorder; cable TV subscription; microwave oven; automatic washing machine; house maid; semi-automatic washing machine; motorcycle; newspaper and/or magazine subscription; computer; internet access; motorcycle; car. The scores were calculated using the principal components method (range: 0–3.39) and were divided into quintiles. Higher scores indicate higher socioeconomic position [6].

The variable self-reported chronic disease included the following illnesses: hypertension, diabetes, asthma, bronchitis, depression, migraine, epilepsy, cancer, chronic digestive disease (ulcer, gastritis), and mental illness (schizophrenia, psychosis, anxiety disorder, bipolar disorder, obsessive-compulsive disorder, panic disorder, anorexia, bulimia). Participants were classified as having a chronic disease if they reported having at least one of the above conditions.

### Statistical analyses

The perceived urban violence score was constructed from variables that assessed the respondents’ insecurity and perception of fear and danger of suffering some form of violence in the neighborhood using exploratory factor analysis. Factor analysis is a data reduction method, which is based on the assumption that highly correlated observed variables (indicators, items, or manifest variables) reflect the action of one or more (unobservable) latent variables or factors [25]. By estimating the latent factors, we were able to account for all or most of the variability generated by the observed variables using a few factors [25, 26]. Because variables were ordinal, we used the polychoric correlation matrix in factor analysis to calculate the scores [26].

Variables with a *p* value ≤ 0.2 on univariate analysis were included in the multivariate analysis. The sampling design was incorporated into the analysis using Stata ‘svy’ command. The strength of the associations was estimated by the odds ratios (OR) and 95% confidence intervals.

To construct the final model, we adopted the hierarchical approach, a sequential process in which the variables entry into the analysis in blocks following the theoretical model presented in Fig. 1 [13]. We use this model to better evaluate how perceived urban violence is associated with health self-assessment, hierarchically adjusting the confounding factors, understanding that they are moderators of this association [27]. Nested models were evaluated using the Wald test. The adjustment of the final model was assessed by the Hosmer Lemeshow test, considering the sampling design.

The evaluated models were: model 1: Urban Violence Perceived Score.

Model 2: model 1 plus years residing in the neighborhood.

Model 3: model 2 plus age, sex (for all participants only) and marital status.

Model 4: model 3 plus schooling and socioeconomic position score.

Model 5: model 4 plus alcohol consumption, smoking, healthy diet and physical activity.

Model 6: model 5 plus satisfied with his/her own weight and reporting of chronic diseases.

All analyses were performed using Stata software version 12.0 (StataCorp, College Station, TX, USA).

## Results

Of the 955 participants, 54.4% were women. Among the women, 79.7% rated their health as very good/good whereas among men this classification was reported by 83.9%. The estimated urban violence score ranged from 1.21 to 4.85 (mean ± SD: 2.32 ± 0.77) for women and 1.21 to 4.65 (2.14 ± 0.70) for men. In addition, 54.8% of the participants were aged 18–24 years, 32.7% were married or living in a stable union: 38,34% of the women and 25,90% of the men.

Nearly three-fourths (73.3%) of the respondents had not completed high school whereas 9.3% of women and 7.1% of men had university graduate/post-undergraduate studies. Regard the SPS quintiles 47.4% of the women were in the two lower quintiles (1 and 2) and 35.5% in the two upper quintiles (4 and 5). For the males the percentages were 34.7% in the two lower quintiles and 42.9% in the upper two quintiles (Table 1).

**Table 1:** Frequency distribution by sex of self-rated health (SRH) and selected variables among 955 young adults (18–29 years). Belo Horizonte Health Study, Brazil, 2008–2009

Variable	Total *N*(%)	Females *N* (%)	Males *N* (%)
Participants	955 (100)	519 (54,35)	436 (45,65)
Self-rated health			
Very good/good	779 (81,70)	413 (79,70)	366 (83,90)
Fair/poor/very poor	175 (18,30)	105 (20,30)	70 (16,10)
Perceived violence score	1,21- 4,85 ($\overline{\mathrm{X}}$:2,23; dp:0,74)	1,21 - 4,85 ($\overline{\mathrm{X}}$:2,32;dp:0,77)	1,21 - 4,65 ($\overline{\mathrm{X}}$:2,14;dp:0,70)
Time residing in the same neighborhood	($\overline{\mathrm{X}}$:13,81;dp:9,02)	($\overline{\mathrm{X}}$:13,05;dp:8,92)	($\overline{\mathrm{X}}$:14,72;dp:9,06)
Age (years):			
18–24	523 (54,80)	268 (51,60)	255 (58,50)
25–29	432 (45,20)	251 (48,40)	181 (41,50)
Marital state			
Married/living together	312 (32,67)	199 (38,34)	113 (25,90)
Single, divorced/separated	643 (67,33)	320 (61,66)	323 (74,10)
Educational level			
Able to read, primary school, 1st-4th grades not completed, 1st-4th grade completed, 5th-8th grade not completed	216 (22,64)	123 (23,70)	93 (22,20)
5th-8th grade completed, high school not completed	483 (50,63)	261 (50,40)	222 (50,10)
High school graduate, technical school, attended university	176 (18,45)	86 (16,60)	90 (20,60)
University graduate, post-undergraduate studies	79 (8,28)	48 (9,30)	31 (7,10)
Socioeconomic position score^a^			
1	213 (22,40)	136 (26,20)	77 (17,70)
2	184 (19,30)	110 (21,20)	74 (17,00)
3	185 (19,40)	89 (17,20)	96 (22,00)
4	193 (20,30)	95 (18,30)	98 (22,50)
5	178 (18,70)	89 (17,20)	89 (20,40)
Alcohol consumption			
Non-drinker	608 (63,67)	378 (72,80)	230 (52,70)
Moderate consumption	111 (11,62)	56 (10,80)	55 (12,60)
Excessive consumption	236 (24,71)	85 (16,40)	151 (34,60)
Smoking			
Yes	154 (16,10)	74(14,30)	80 (18,40)
No	801 (83,90)	445 (85,70)	356 (81,60)
Satisfied with his/her own weight			
Yes	417 (43,70)	194 (37,38)	223 (51,20)
No	538 (57,30)	325 (62,62)	213 (48,80)
Physical activity			
Yes	376 (39,40)	134 (25,80)	242 (55,50)
No	579 (60,60)	385 (74,20)	194 (44,50)
Healthy diet			
Yes	314 (32,90)	185 (35,65)	129 (29,60)
No	641 (67,10)	334 (64,35)	307 (70,40)
Chronic disease			
Yes	339 (35,53)	233 (44,89)	106 (24,37)
No	615 (64,47)	286 (55,11)	329 (75,63)

Missing data: chronic disease (*n* = 7), SRH (*n* = 1), education level (n = 1), and socioeconomic score (*n* = 2). ^a^. SPS: Score ranging from 0 to 3.39; higher number of assets = higher score

Roughly 36.3% of respondents reported regular alcohol consumption and 34.6% of the men and 16.4% of the women reported excessive alcohol consumption (≥ three times a week or > five drinks per day). 18.4% of men and 14.3% of women were smokers.

Among women, 62.6% reported not being satisfied with their weight, 74.2% did not exercise regularly and 64.4% did not have a healthy diet. For the men whereas 48.8% were not satisfied with their weight, 44.5% were not physical active, and 70.4% did not have a healthy diet. The presence of at least one chronic disease was reported by 35.5% of respondents, of which 44,9% were women and 24,4% were men.

Over fifty (62.5%) of respondents reported residing in the same neighborhood for over 10 years, 58.8% of women and 67.0% of men (Table 1).

Table 2 shows the results of univariate analysis by sex. Among women, fair poor/very poor SRH was associated with age between 25 and 29 years, be married or living in a stable union, low SPS, being dissatisfied with their weight, not exercising regularly, not having a healthy diet, and having some chronic disease.

**Table 2:** Odds ratio and confidence intervals for the sex-specific association between selected variables and self-rated health (SRH) among 955 young adults (18–29 years). Belo Horizonte Health Study (BHS), Belo Horizonte, Brazil, 2008 -2009. Univariate analysis

Variable	SRH Females OR (95% CI)	SRH Males OR (95% CI)
Perceived violence score	1,46 (0,98-2,17)	1,27 (0,79-2,07)
Time residing in the same neighborhood	0,98 (0,95-1,02)	1,00 (0,97-1,04)
Age (years):		
18–24	1,00	1,00
25–29	0,54 (0,31-0,96)	0,74 (0,38-1,44)
Marital state		
Single, divorced/separated	1,00	1,00
Married/living together	1,93 (1,10-3,51)	1,74 (0,93-3,30)
Educational level		
Able to read, primary school, 1st-4th grades not completed, 1st-4th grade completed, 5th-8th grade not completed	1,00	1,00
5th-8th grade completed, high school not completed	0,66 (0,33-1,33)	0,52 (0,24-1,12)
High school graduate, technical school, attended university	0,55 (0,23-1,26)	0,51 (0,19-1370
University graduate, post-undergraduate studies	0,04 (0,01-0,18)	0,71 (0,11-4,54)
Socioeconomic position score^a^		
1	1,00	1,00
2	0,46 (0,23-0,93)	0,62 (0,02-1,68)
3	0,29 (0,13-0,66)	0,44 (0,18-1,10)
4	0,27 (0,11-0,63)	0,23 (0,08-0,63)
5	0,15 (0,05-0,45)	0,43 (0,15-1,21)
Alcohol consumption		
No	1,00	1,00
Yes	1,05 (0,73-1,51)	1,24 (0,85-1,80)
Smoking		
No	1,00	1,00
Yes	1,41 (0,65-3,03)	3,81 (1,74-8,35)
Healthy diet		
No	1,00	1,00
Yes	0,35 (0,18-0,69)	0,43(0,20-0,94)
Satisfied with his/her own weight		
No	1,00	1,00
Yes	0,40 (0,21-0,77)	0,39 (0,19-0,77)
Physical activity		
No	1,00	1,00
Yes	0,51 (0,27-0,96)	0,41 (0,20-0,84)
Chronic disease		
No	1,00	1,00
Yes	2,16 (1,24-3,77)	4,10 (1,98-8,50)

^a^ Socioeconomic position score score ranging from 0-3.39: highest number of assets = highest score

Men who rated their health as fair/poor/very poor more frequently smoked, were dissatisfied with their weight, did not exercise regularly, consumed fewer fruits and vegetables, and had some chronic disease compared to men who rated their health as very good/good.

In Table 3 are shown each of the hierarchical models. For all participants and males, after accounting for all potential confounding variables (Model 6), perceived urban violence was not associated with SRH. For the women the hierarchical models from 3 to 6 shows association between perceived violence in the neighborhood and fair/poor/very poor SRH. An unit increase in the perceived violence in the neighborhood score increased by 52% the odds of women rating their health as fair, poor or very poor, after accounting for all potential confounding variable (OR = 1.52; 95% CI: 1.04–2.21, Model 6).

**Table 3:** Perceived urban violence on health self-assessment in each of the hierarchical models for all, females and males participants for 955 young adults (18-29 years). Belo Horizonte Health Study, Belo Horizonte, Brazil, 2008–2009

Models	SRH Total OR (95% CI)	SRH Females OR (95% CI)	SRH Males OR (95% CI)
Model 1	1,40 (1,05-1,87)	1,46 (0,99-2,16)	1,27 (0,79-2,03)
Model 2	1,37 (1,02-1,84)	1,47 (1,00-2,17)	1,15 (0,72-1,83)
Model 3	1,38 (1,03-1,86)	1,54 (1,06-2,25)	1,15 (0,71-1,85)
Model 4	1,28 (0,96-1,71)	1,47 (1,04-2,09)	1,05 (0,64-1,73)
Model 5	1,27 (0,94-1,72)	1,47 (1,02-2,12)	1,02 (0,59-1,75)
Model 6	1,28 (0,94-1,74)	1,52 (1,04-2,21)	0,94 (0,52-1,68)

Model 1: Urban Violence Perceived Score

Model 2: Model 1 plus years residing in the neighborhood

Model 3: Model 2 plus age, sex (for all participants only) and marital status

Model 4: Model 3 plus schooling and socioeconomic position score

Model 5: Model 4 plus alcohol consumption, smoking, healthy diet and physical activity

Model 6: Model 5 plus satisfied with his/her own weight and reporting of chronic diseases

## Discussion

Our results indicate that perceived violence in the neighborhood is associated with fair/poor/very poor SRH in young women; however, we did not find the same association for young males.

Urban violence is a phenomenon that demands a multifaceted, inter-sectorial, and interdisciplinary approach, related to individuals, groups, classes, and institutions, which in their relations employ different methods and means of coercion and annihilation of people. Living in large cities has implications for people’s lives and on the social determinants that operate through various process [13, 28].

Violence, increased neighborhood crime, and weaker social cohesion are also dynamically linked to the characteristics of the physical and social environment, increasing stress levels and changing people’s behavior, leading to worsening health and poor SRH. Thus, both physical and social environment as well as behavioral characteristics can weaken social ties and increase violence [13, 14, 16, 19].

The magnitude, nature, and impact of urban violence on health differ greatly for men and women. Violence against women has been associated with worse SRH, worse quality of life, gynecological symptoms, depression, chronic pain, post traumatic stress disorder and substance abuse [28–30]. A 2006 Swedish study evaluated 34,707 women in two age groups: 18–29 years and 30–44 years, with results similar to our study, reporting an association between the risk of some form of violence in the neighborhood and worst SRH in both age groups. This study also found synergistic effect between violence and low socioeconomic status worsening the self-assessment of health [29].

Research evidence suggests that gender based violence can be concentrated at the neighborhood level, especially in disadvantaged, vulnerable urban settings. Disadvantaged urban settings can exacerbate underlying gender-based power disparities, subjecting young women to intensive harassment, pressure for early sexual activity, and a pervasive threat of sexual and physical violence [30]. Large populated neighborhoods characterized by weak social ties and low collective efficacy can also increase the risk of violence [29–31].

There are possible sociological explanations for the results observed in our study. Some studies argue that women are taught, from an early age, to take care of people and family and to be more empathetic towards community suffering, whereas men are encouraged to be aggressive and competitive [31, 32]. These differences in the socialization process predispose women to internalize their difficulties, resulting in an increased incidence of depression, anxiety, and possibly poor self-rating of health. Conversely, men exposed to community violence tend to develop aggressive behaviors and are more prone to crime [31, 32].

No association between perceived urban violence and SRH may also reflect adaptation by young men growing up exposed to neighborhood violence [32]. Some researchers suggest that male youths who are chronically exposed to community violence may become desensitized and suppress feelings of sadness or anxiety [33, 34]. Male youths may develop initial internalizing symptoms in reaction to new or unusual exposure to violence, but over time their symptoms might be expected to abate [33]. Thus, rather than concluding that young males do not experience symptoms or that they minimize their response to a violent event, it is possible that there are fundamental gender differences in the type of response [34]. It appears that moderating factors may mitigate the conditions under which violence exposure in youth leads to adverse outcomes [35].

The Belo Horizonte Health Study was not originally designed to specifically investigate young adults. Although consistent with the literature, our study had not found an association between perceived violence in the neighborhood and poor self-reported health among young men. We cannot rule out the study sample size and low power to estimate this association. However, this study is of great relevance because it was the first study to evaluate the association between sex-specific perception of violence in the neighborhood and SRH among young adults in a middle income country.

This study has several strengths. Few studies have examined the association between perceived urban violence and SRH among young adults, despite the relevance of the issue especially when violence is one of the leading causes of death among young people in many countries. Several steps were taken to avoid potential biases, including reliability assessment of the instruments used, use of standard procedures and equipment, extensive training of field personnel in addition to intensive activities with the community to encourage participation in the study. Thus, the internal validity and quality of information were ensured [24].

## Conclusions

We showed that perceived violence in the neighborhood was associated with poor SRH in women, even after adjusting for several individual attributes. Even though the mechanisms responsible for this association have not been clearly elucidated, the results of this study indicate that public and health policies should implement interventions on the physical and social environment of the district or neighborhood that improve the perception of safety and have a positive impact on people’s health, especially women.
